# Nramp1 and NrampB Contribute to Resistance against *Francisella* in *Dictyostelium*

**DOI:** 10.3389/fcimb.2017.00282

**Published:** 2017-06-21

**Authors:** Yannick Brenz, Denise Ohnezeit, Hanne C. Winther-Larsen, Monica Hagedorn

**Affiliations:** ^1^Department of Parasitology, Bernhard Nocht Institute for Tropical MedicineHamburg, Germany; ^2^Institute for Medical Microbiology, Hygiene and Virology, University Medical Center Hamburg-EppendorfHamburg, Germany; ^3^Centre for Integrative Microbial Evolution and Department of Pharmaceutical Biosciences, University of OsloOslo, Norway; ^4^Department of Life Sciences and Chemistry, Jacobs UniversityBremen, Germany

**Keywords:** *Dictyostelium*, *Francisella*, infection, iron transporter, Nramp

## Abstract

The *Francisella* genus comprises highly pathogenic bacteria that can cause fatal disease in their vertebrate and invertebrate hosts including humans. In general, *Francisella* growth depends on iron availability, hence, iron homeostasis must be tightly regulated during *Francisella* infection. We used the system of the professional phagocyte *Dictyostelium* and the fish pathogen *F. noatunensis* subsp. *noatunensis* (*F.n.n*.) to investigate the role of the host cell iron transporters Nramp (natural resistance associated macrophage proteins) during *Francisella* infection. Like its mammalian ortholog, *Dictyostelium* Nramp1 transports iron from the phagosome into the cytosol, whereas the paralog NrampB is located on the contractile vacuole and controls, together with Nramp1, the cellular iron homeostasis. In *Dictyostelium*, Nramp1 localized to the *F.n.n*.-phagosome but disappeared from the compartment dependent on the presence of IglC, an established *Francisella* virulence factor. In the absence of Nramp transporters the bacteria translocated more efficiently from the phagosome into the host cell cytosol, its replicative niche. Increased escape rates coincided with increased proteolytic activity in bead-containing phagosomes indicating a role of the Nramp transporters for phagosomal maturation. In the *nramp* mutants, a higher bacterial load was observed in the replicative phase compared to wild-type host cells. Upon bacterial access to the cytosol of wt cells, mRNA levels of bacterial iron uptake factors were transiently upregulated. Decreased iron levels in the *nramp* mutants were compensated by a prolonged upregulation of the iron scavenging system. These results show that Nramps contribute to host cell immunity against *Francisella* infection by influencing the translocation efficiency from the phagosome to the cytosol but not by restricting access to nutritional iron in the cytosol.

## Introduction

Iron is essential for growth of virtually all organisms and is an important cofactor in many redox reactions. On the other hand, phagocytic cells can also use iron to act as a cofactor for generation of antimicrobial radicals. Therefore, host cells and intracellular bacterial pathogens are in a constant struggle for this nutrient. Consequently, iron uptake and homeostasis during infection is tightly regulated by both the host and the pathogen (Schaible and Kaufmann, 2004).

Members of the natural resistance associated macrophage protein (Nramp) family play an important regulatory role in cellular iron homeostasis. Nramps are transmembrane transporters of divalent metal ions, especially Fe^2+^, Mn^2+^, and Zn^2+^, and widely distributed in prokaryotes and eukaryotes (Cellier et al., 1995; Courville et al., 2006; Nevo and Nelson, 2006). In mammals, two Nramp family members are present, Nramp1 (SLC11A1) and DMT-1 (SLC11A2 or Nramp2). Nramp1 is found on endosomal and lysosomal vesicles as well as phagosomal membranes in macrophages (Gruenheid et al., 1997; Searle et al., 1998), where it transports Fe^2+^ and Mn^2+^ from the phagosome into the cytosol dependent on a proton gradient (Buracco et al., 2015). Nramp1 was identified as part of the *Bcg*/*Ity*/*Lsh* locus in mice, which contributes to natural resistance against intracellular pathogens such as *Mycobacteria, Leishmania*, and *Salmonella* (Vidal et al., 1993, 1995). In humans, polymorphic variations of the *nramp1* gene are linked to tuberculosis (Wu et al., 2013), leprosy (Abel et al., 1998) and oropharyngeal tularemia (Somuk et al., 2016). In contrast, isoforms of DMT-1 are responsible for transferrin-independent iron uptake (Canonne-Hergaux et al., 1999) or recycling via endosomes (Gruenheid et al., 1999; Touret et al., 2003), and have been associated with microcytic anemia (Fleming et al., 1997; Canonne-Hergaux et al., 2000) and neurodegeneration (Salazar et al., 2008).

*Dictyostelium* discoideum is an amoeba, which is frequently used to dissect basic cellular processes (Muller-Taubenberger et al., 2013). In nature, *Dictyostelium* thrives on bacteria as a motile, single cell organism. However, when food is scarce, many amoebae aggregate to form a true multicellular organism that evolves further into a fruiting body harboring stress-resistant spores. The cycle closes when the spores are exposed to nutrients and single cells hatch from the spores. Most important for our study, at the single cell-stage, the amoeba is a professional phagocyte, and represents many features with cells of the innate immune system (Bozzaro, 2013; Zhang et al., 2016). Like macrophages, *Dictyostelium* can be infected with various pathogenic bacteria and is an established cellular infection model due to its homology to mammalian phagocytes, genetic tools and easy cultivation in the laboratory (Bozzaro and Eichinger, 2011). The genome of *Dictyostelium* comprises two *nramp* genes, *nramp1* and *nrampB*. Similar to its mammalian ortholog Nramp1 is localized on endolysosomal vesicles and is recruited to phagosomes and macropinosomes (Peracino et al., 2006). In contrast, NrampB is closer related to Nramp proteins from protists and fungi and the manganese transporters of proteobacteria. NrampB is localized at the contractile vacuole of the amoeba, a tubular network for the regulation of the cellular osmolarity, where it controls, synergistically with Nramp1, the cellular iron content (Peracino et al., 2013). Both Nramp1 and NrampB affect the replication of vacuole-dwelling bacteria in the amoeba as *Dictyostelium* knockout cell lines of either Nramp are more susceptible to *Legionella* and, in case of Nramp1, to *Mycobacteria* (Peracino et al., 2006, 2010, 2013).

The intracellular bacterium *Francisella tularensis* infects multiple host organisms of both invertebrate and vertebrate origin, and is the causative agent of potentially fatal tularemia in humans (Keim et al., 2007; Foley and Nieto, 2010). Within host cells, infection by *F. tularensis* shows a biphasic course with the bacteria initially residing in a phagosome, which is followed by translocation and a replicative stage in the host cell cytosol (Golovliov et al., 2003; Clemens et al., 2004, 2009; Chong et al., 2008). As shown for other intracellular bacteria, *Francisella* growth depends highly on bioavailable iron in the host cell (Perez and Ramakrishnan, 2014; Perez et al., 2016), but iron also contributes to H_2_O_2_-induced killing of *Francisella* (Lindgren et al., 2011). In contrast to vacuolar pathogens, little is known about the role of iron regulatory Nramp for *Francisella* and other cytosol-dwelling bacteria on the cellular level.

Iron acquisition in *F. tularensis* includes two uptake systems: the *Francisella* siderophore locus (*fsl*) system for ferric iron (Fe^3+^) and the *feo* system for ferrous iron (Fe^2+^) (Perez et al., 2016). Metabolically competent *Francisella* species including *F. noatunensis* subsp. *noatunensis* (*F.n.n*.) express the *feo*-factors FeoA and FeoB but only one protein (IucA/C) for Fe^3+^ uptake (Sridhar et al., 2012).

In this study, we used the established *Dictyostelium*/*F.n.n*. infection system (Lampe et al., 2016) to investigate the role of Nramp1 and NrampB during the infection with *Francisella*. We determined the localization of both Nramps during infection with *F.n.n*. and quantified the intracellular growth of the bacteria in the absence of the iron transporters. The influence of Nramp1 and NrampB on the phagosomal and cytosolic stages was determined by quantifying phagosomal escape of *F.n.n*. and bacterial mRNA levels of iron acquisition factors during infection. Our results suggest that Nramps contribute to resistance against *Francisella* infection by influencing the phagosomal stage of the bacteria rather than nutritional stress.

## Materials and methods

### Cells, bacterial strains, and culture conditions

*Dictyostelium* discoideum cells (Ax2) were cultured adherently in axenic HL5-C medium supplemented with 100 μg/ml Pen/Strep (Hagedorn and Soldati, 2007). Prof. Salvatore Bozzaro (University of Torino, Italy) kindly provided confirmed knockout cell lines of Nramp1, NrampB and Nramp1/B (Peracino et al., 2006, 2013). Knockout cell lines had been generated by the authors (Peracino et al., 2006, 2013) using homologous recombination to replace the respective gene with the respective coding sequence disrupted by a blasticidin resistance cassette. Ax2 cells expressing Nramp1-GFP and NrampB-GFP were obtained by transformation with the plasmids pDEX-Nramp1::GFPC and pDEX-NrampB::GFPC, respectively [provided by Prof. Bozzaro, (Peracino et al., 2006, 2013)] and grown with 10–30 μg/ml G418. Green (pKK289Km:*gfp*) and red (pKK289Km:*mCherry*) fluorescent *F.n.n*. wild-type and Δ*iglC* bacteria were cultivated in Eugon Broth (EB) shaking culture (100 rpm) at 22°C supplemented with 2 mM FeCl_3_ and 15 μg/ml Kanamycin (Lampe et al., 2016). The in-frame knockout of *iglC* was achieved via a suicide plasmid (pDMK2) containing the fused flanking regions (~1,100 bp each) of *iglC* and a kanamycin resistance according to Lampe et al. (2016) and similar to other *Francisella* species (Lindgren et al., 2009). Results were verified by sequencing and qPCR confirmed no effects on neighboring genes *iglB* and *iglD* (Lampe et al., 2016). Iron depletion during *F.n.n*. cultivation was performed at 22°C using 100 μM 2,2′-dipyridyl (DP, Sigma-Aldrich) in EB as described by Brudal et al. (2013). In short, bacteria were incubated for 48 h until OD 1.5–2, diluted to OD 0.1 and grown for 24 h without FeCl_3_. Subsequently, remaining iron was washed off with PBS (2x) and EB (+DP), the culture was diluted to OD 0.5 and grown for 24 h until OD 1.

### Infection assay

The infection of *Dictyostelium* cells with *F.n.n*. was performed as described (Lampe et al., 2016). *F.n.n*. bacteria were grown at 22°C in Eugon Broth supplemented with 2 mM FeCl3 and 15 μg/ml Kanamycin until reaching the exponential growth phase. At least 24 h prior to infection, *Dictyostelium* cells were grown at 22°C in HL5-c medium without antibiotics in 25 or 75 cm^2^ cell culture flasks. On infection day, *F.n.n*. equivalent to MOI 60 were centrifuged on a *Dictyostelium* monolayer of 80–100% confluency at 100 × g and 21°C for 30 min. After 5 min of additional time for phagocytosis, free bacteria were washed off with HL5-C without antibiotics and checked visually for remaining extracellular bacteria. Infected cells were seeded in HL5-C without antibiotics in 10 cm culture dishes at respective cell numbers (6 h post infection (hpi): 1 × 10^7^, 24 hpi: 4.5 × 10^6^, 48 hpi: 2 × 10^6^) and cultivated adherently at 22°C. At each timepoint, cells were resuspended, counted with a CASY Cell Counter and ~1 × 10^6^ cell were fixed with 4% paraformaldehyde (PFA) in Sørensen buffer for subsequent antibody staining and analysis via fluorescent microscopy or flow cytometry. For flow cytometry analysis, at least 4 × 10^5^ cells were quantified per sample using a FACSCalibur flow cytometer (Becton Dickinson) and analyzed according to Lampe et al. (2016) via FlowJo software v10. In short, dead cells and extracellular *F.n.n*. were excluded from analysis by gating only on living *Dictyostelium* cells using FSC/SSC. Infected and non-infected *Dictyostelium* populations were separated using SSC plotted as a function of green fluorescence (FL-1). The green fluorescence of intracellular *F.n.n*. was expressed as relative fluorescence units (RFU) per volume unit of cell culture (RFU/ml) as followed: the infection rate was multiplied with *Dictyostelium* cell number/ml to obtain infected cells/ml. This value was multiplied with the mean FL-1 value of *F.n.n*. only, which was caluculated as mean FL-1 of infected cells - mean FL-1 of non-infected cells. All samples were analyzed with the same fluorescence detector settings. The resulting RFU/ml correlate with *F.n.n*. genome equivalents/ml as shown by Lampe et al. (2016).

### Immunohistochemistry

Immunolabeling of PFA-fixed samples for microscopy (Hagedorn et al., 2006) and flow cytometry (Lampe et al., 2016) was performed as described. Monoclonal antibody against the putative copper transporter and endosomal marker p80 (Ravanel et al., 2001) was obtained from P. Cosson (University of Geneva, Switzerland) and used 1:10 in blocking solution (0.5% FCS, 0.1% Triton in PBS). Rabbit polyclonal GFP-antibody (MBL International) was used at a dilution of 1:1,000 for microscopy and flow cytometry. Anti-rabbit and anti-mouse secondary antibodies from goat (Invitrogen) were coupled to AlexaFluor 488 and 568, respectively, and diluted 1:1,000. For quantitative microscopic analysis, a minimum of 100 bacteria was analyzed at each time-point. For statistical analysis of multiple groups to the wt control group over time, a repeated measures one-way ANOVA followed by Dunnett's *post-hoc* test was performed for each timepoint using Prism 7.0c software.

### Microscopy

For live cell imaging, infected cells were resuspended in filtrated HL5-C and seeded in μ-Slide 8 well chambers (Ibidi) shortly before imaging. Microscopic analysis was performed at 21°C using a Zeiss LSM5 Live confocal microscope with a 100x Europlan apochromat oil immersion objective (N.A. 1.4) and a Diode-Laser 488, as well as a DPSS-Laser 561 [single track mode, 1 Airy unit, dual-band filter (500–545 band pass, 575 long pass)]. Image brightness and contrast were adjusted with ImageJ (Schneider et al., 2012) to whole images. A minimum of 100 bacteria were quantified at each timepoint except for *F.n.n*. Δ*iglC* at 2 hpi (50 bacteria). Imaging of PFA-fixed samples was performed with an Olympus IX81 confocal microscope equipped with an Olympus 100x UPlanSApo oil immersion objective (N.A. 1.4).

### qRT-PCR of *F.n.n*. iron accumulation genes

*F.n.n*. infected *Dictyostelium* cells or cultured bacteria were lysed with Trizol (Life Technologies). RNA extraction was performed using the PureLink RNA Mini Kit (Life Technologies) according to the manufacturer's instructions for Trizol extraction. For cDNA synthesis, the Maxima First Strand cDNA Synthesis Kit (Thermo Scientific) was applied according to the manufacturer's instructions. One microliter of 1:10 diluted cDNA was used for each sample as a template in a 10 μl reaction volume using LightCycler® 480 SYBR Green I Master mix (Roche) and a Rotor-Gene RG-3000 qPCR machine (Corbett Research). The thermal cycle conditions were as follows: 1 cycle at 95°C for 5 min; 40 cycles of amplification at 95°C for 30 s, 60°C for 30 s and 72°C for 30 s. A melting curve analysis was performed from 67–95°C with steps of 1°C. Samples of three infection experiments were run in duplicates. Gene-specific primer sequences of iron related genes (*feoA, feoB, iucA/C*) and reference genes (*ftsZ, polA, fopA*; Brudal et al., 2013) were designed with Primer3 software using contig data of *F.n.n*. (Sjodin et al., 2012) as a template and obtained from Brudal et al. (2013), respectively (Table S1). Results were analyzed using the ΔΔCt method and tested for statistical significance with a repeated measures one-way ANOVA followed by Dunnett's post test using the Prism 7.0c software.

### Quantification of proteolysis and pH in bead phagosomes

The phagosomal proteolysis and pH of *Dictostelium* cell lines was quantified using fluorescently-labeled, 3 μm silica beads according to Sattler et al. (2013) with minor adaptations. In short, latex beads were labeled with 0.15 mg of either DQ Green BSA (proteolysis-sensitive) or FITC (pH-sensitive) and Alexa 568 succinimidyl ester as a reference dye. Fluorescently labeled beads were added at a bead/cell ratio of 2:1 to a 90–100% confluent monolayer of *Dictyostelium* cells in a 96 well cell culture microplate μClear/black (Greiner). After short centrifugation (10 s, 300 × g, 22°C), cells were left 1 min for phagocytosis. Free beads were washed off and the fluorescence emission of intracellular beads was measured every 2 min for 2 h at 535 and 590 nm using an Infinite F200 fluorescence reader (Tecan). Filter settings for excitation/emission were as followed: 485(20)/535(25) for DQ Green/FITC and 550(10)/590(20) for Alexa568. To calculate the intraphagosomal pH from the 535/590 nm emission ratio, a calibration curve was prepared for each experiment using reference pH buffers in a range of pH 3 to 7. For comparison of the acidification and proteolysis profiles, linear regression lines were calculated for the timeframes of interest (proteolysis: linear range; covering ~25 datapoints each, acidification: *t* = 0 min to the minimal turning point (mtp), reneutralisation: mtp to *t* = 120 min; Figure S4) and the slopes were tested for significance using a one-way ANOVA followed by Dunnett's multiple comparison post test using the Prism 7.0c software.

### Survival test of exocytosed *F.n.n*.

*Dictyostelium* cells infected with either *F.n.n*. wt and Δ*iglC* were detached at 6 hpi and 1 ml cell culture including exocytosed bacteria was centrifuged at 500 × g for 3 min to pellet cells but not bacteria. The supernatant was centrifuged at 5,000 × g for 5 min and the bacterial pellet was dissolved in 100 μl HL5-C medium. 20 μl of undiluted and 1:50 diluted bacterial suspension was dropped on a chocolate agar plate and incubated for 1 week.

## Results

### Nramp1 but not NrampB associates with *F.n.n*.-containing phagosomes

**Figure 1:**
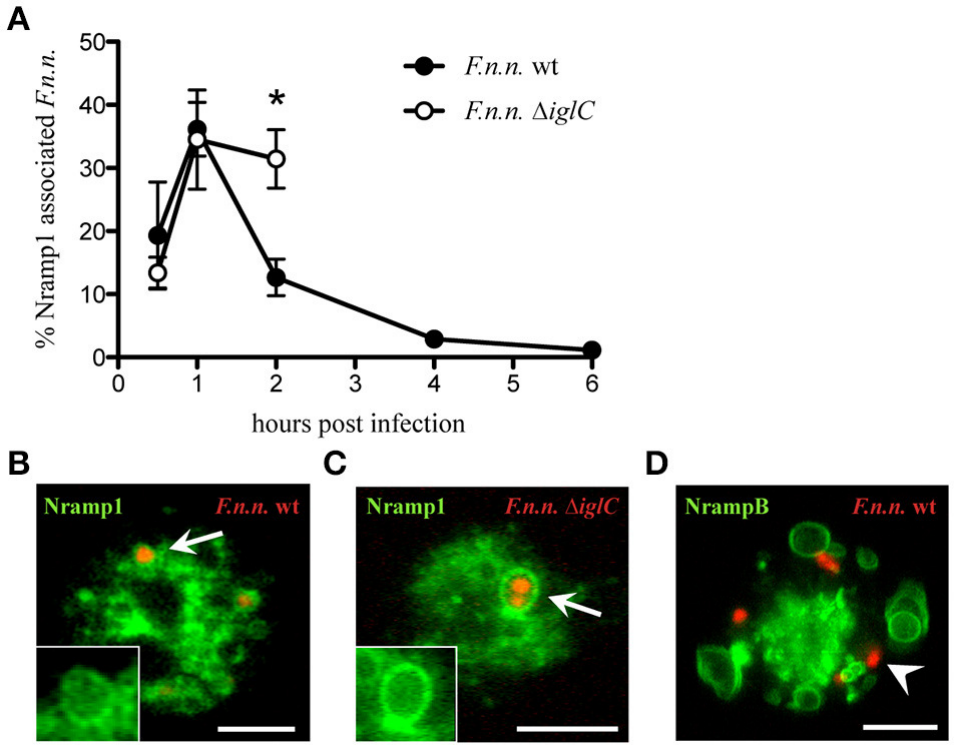
Association of Nramp1 and NrampB with the *F.n.n*.-phagosome. **(A–C)** Nramp1-GFP expressing cells were infected with *F.n.n*. wt and Δ*iglC* expressing mCherry and monitored via life cell microscopy. Shown are the means ± SEM of association rates over 6 h of 3–4 experiments **(A)** and representative micrographs of positive association at 1 hpi **(B,C)**. The inlet displays the green channel of the region of interest. **(D)** Representative image of NrampB-GFP infected with *F.n.n*. wt mCherry at 1 hpi. Arrow: Nramp1-positive, arrowhead: NrampB-negative. Scale bar: 5 μm. Unpaired two-tailed Student's *t*-test: ^*^p < 0.05.

As transmembrane transporters of divalent metal ions, Nramp1 and NrampB change the ion composition of the compartment on which they are located. Thus, Nramp transporters could directly modify the environment of vacuolar bacteria. To investigate if Nramp1 and NrampB localize to the *F.n.n*.-phagosome (FP) in *Dictyostelium* cells, we infected amoeba expressing Nramp-GFP-fusion proteins with *F.n.n*. wild-type (wt) bacteria expressing mCherry. In addition, to monitor whether Nramp recruitment was actively manipulated by virulent *F.n.n*., we compared Nramp1 association with wt bacteria as well an avirulent mutant strain lacking a major component of the pathogenicity island IglC (*F.n.n*. Δ*iglC*). The proportion of Nramp-GFP associated FPs was quantified via life cell imaging in the early phase of infection (1–6 hpi). Until 1 hpi, wt and Δ*iglC* bacteria share similar association rates with Nramp1 at the FP (Figures 1A–C). However, whilst the association of wt *F.n.n*. with the iron transporter drops at 2 hpi, the avirulent mutant remains in an Nramp1-positive compartment. However, at 4 hpi, most *F.n.n*. Δ*iglC* are killed and exocytosed (Figure S1; Lampe et al., 2016) and not enough bacteria were available for reliable quantification. In contrast, wt *F.n.n*. were not observed in NrampB-positive compartments at any time post infection (Figure 1D).

### High bacterial load in *nramp* knockout cells

The localization of Nramp1 on the FP and the synergistic regulation of intracellular iron homeostasis by both Nramps (Peracino et al., 2013) led us to investigate the functional impact of Nramp1 and NrampB on *F.n.n*. infection. We infected *Dictyostelium* Δ*nramp1*, Δ*nrampB* and Δ*nramp1/B* cells with GFP-expressing *F.n.n*. wt bacteria and compared the course of infection to *Dictyostelium* wt cells. The bacterial load was then monitored until 48 hpi quantifying bacteria by both fluorescence microscopy and flow cytometry.

**Figure 2:**
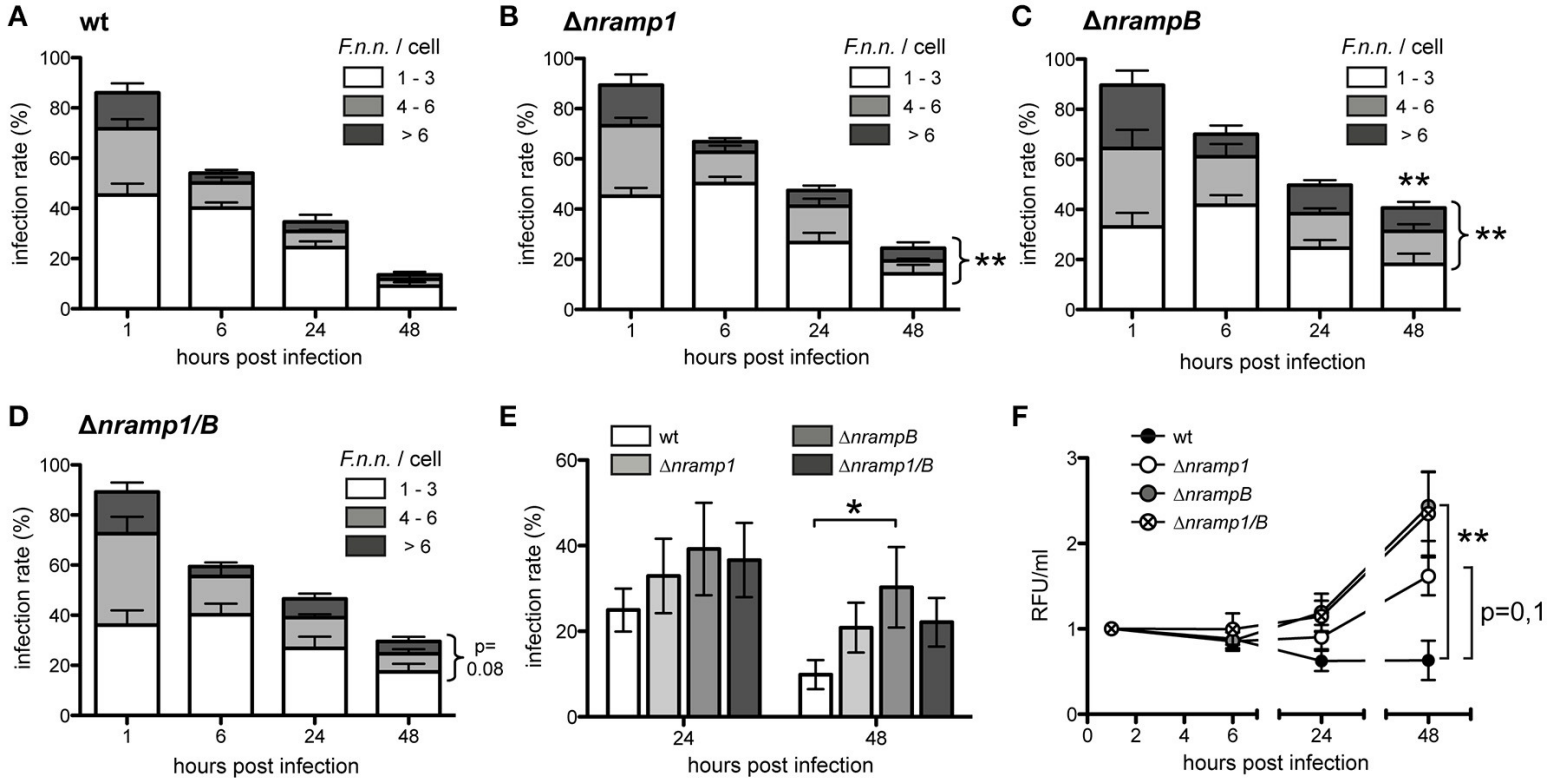
Increased *F.n.n*. growth in the absence of Nramp1 and NrampB. **(A–D)** Infection rate and bacteria/cell of *Dictyostelium* wt **(A)**, Δ*nramp1* **(B)**, Δ*nrampB* **(C)** and Δ*nramp1/B* **(D)** cells infected with *F.n.n*. wt expressing GFP over 48 hpi. Number of *F.n.n*./cell: 1–3 (white), 4–6 (gray), >6 (black). Shown are differences of the mean (±SEM, *n* = 5) analyzed for each timepoint with a repeated measures one-way ANOVA with Dunnett's post test: ^*^p < 0.05, ^**^p < 0.01. **(E)** Infection rate of wt and *nramp* mutant cells infected with *F.n.n*. wt GFP at 24 and 48 hpi monitored via flow cytometry (mean ±SEM, *n* = 7). **(F)** Relative bacterial fluorescence/ml in *Dictyostelium* wt and *nramp* knockout cells over 48 hpi (mean ±SEM, *n* = 7). **(E,F)** Repeated measures one-way ANOVA with Dunnett's *post-hoc* test for each timepoint: ^*^p < 0.05, ^**^p < 0.01.

Quantification of individual bacteria per cell by fluorescence microscopy showed a similar uptake of bacteria by wt and *nramp* knockout *Dictyostelium* cells (Figures 2A–D). However, from 24 hpi, the bacterial load per *Dictyostelium* cell was higher in the *nramp* deletion mutants. At 48 hpi, *nramp* mutants showed higher overall infection rates (wt: 13.6 ± 3.8%, Δ*nramp1*: 24.5 ± 1.9%, Δ*nrampB*: 40.7 ± 7.8%, Δ*nramp1/B*: 29.6 ± 3.5%) and increased proportions of highly infected cells (more than 3 bacteria: wt: 20.4 ± 12.5%, Δ*nramp1*: 51.1 ± 8.8%, Δ*nrampB*: 57.4 ± 6.7%, Δ*nramp1/B*: 41.3 ± 9.9%). The bacterial burden with more than 3 bacteria per cell over time is significantly higher in the *nramp* mutants indicating more bacterial growth at the single cell level.

To apply another approach to quantify *F.n.n*. growth we used quantitative flow cytometry and measured infection rate and bulk fluorescence of intracellular bacteria per volume over 48 h. Overall, the flow cytometry analysis recapitulated our results obtained by fluorescence microscopy. The infection rates of the *Dictyostelium nramp* mutants (Δ*nramp1*: 20.84 ± 5.8%, Δ*nrampB*: 30.3 ± 9.4%, Δ*nramp1/B*: 22.1 ± 5.7%) were higher in comparison to wt cells (9.9 ± 3.4%) at 48 hpi (Figure 2E). In principal, *Dictyostelium* cell growth can have an impact on the infection rate. However, the growth rates of the different cell lines were comparable over 48 h and could therefore not account for differing infection rates (Figure S2). The quantification of bacterial fluorescence (relative fluorescence units/ml) also showed higher bacterial loads in the mutant cell lines compared to wt cells (Figure 2F). This suggests that Nramp1 and NrampB contribute to resistance of the amoeboid host cell against *F.n.n*. growth.

### *F.n.n*. escapes the phagosome more efficiently in the absence of Nramp transporters

**Figure 3:**
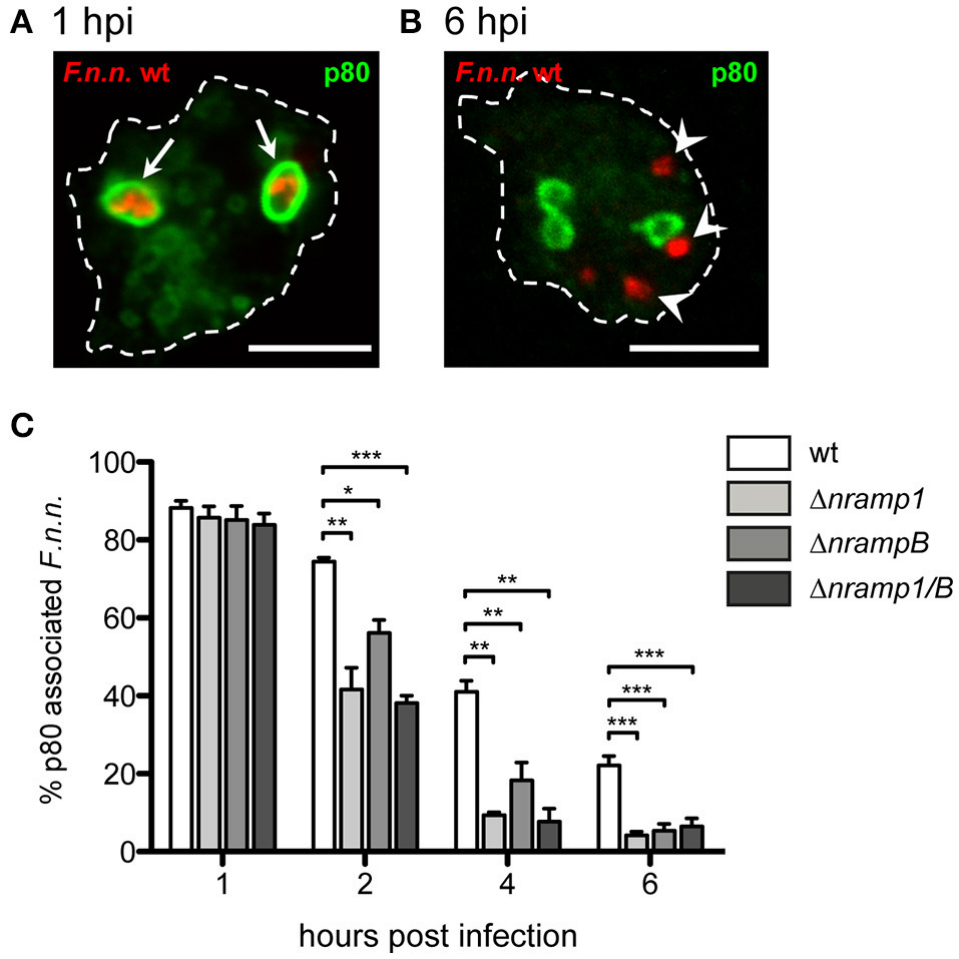
Phagosomal escape of *F.n.n*. in *nramp* knockout cells. **(A,B)** Representative micrographs of p80-positive and –negative *F.n.n*. in *Dictyostelium* wt cells at 1 and 6 hpi, respectively. Arrows indicate p80-positive F.n.n., arrowheads indicate p80-negative F.n.n. **(C)** *Dictyostelium* wt, Δ*nramp1*, Δ*nrampB*, and Δ*nramp1/B* cells were infected with *F.n.n*. wt GFP and the association of the endosomal marker p80 with the FP was monitored over 6 hpi by fluorescence microscopy (mean ± SEM, *n* = 3–6). Statistical analysis was performed applying a repeated measures one-way ANOVA analysis with Dunnett's *post-hoc* test for each timepoint: ^*^p < 0.05, ^**^p < 0.01, ^***^p < 0.001.

It seems most likely that Nramp1 and NrampB control bacterial replication by affecting the phagosomal stage of *F.n.n*. infection. Like *F. tularensis, F.n.n*. escapes the p80-positive phagosome in the late phagosomal stage in *Dictyostelium* (Lampe et al., 2016). Therefore, the endosomal protein p80, a putative copper transporter (Ravanel et al., 2001), was applied as a marker for intraphagosomal vs. cytosolic bacteria phagosomal membranes (Figures 3A,B).

At 1 hpi, more than 80% of *F.n.n*. were localized in phagosomes of wt and *nramp* knockout cells (Figure 3C). However, starting at 2 hpi, significantly less bacteria were located in p80-positive compartments in the *nramp* deletion mutants with a comparable total load of bacteria per cell between wild-type and mutant cells. This suggests that more bacteria translocate into the cytosol and reach the replicative, cytosolic phase in the *nramp* mutants, which results in a higher bacterial load per cell in the mutant strains at the late infection phase. Even though not statistically significant, in comparison to the Δ*nrampB* strain at 2 and 4 hpi, bacteria seem to escape more efficiently in the Δ*nramp1* cells. This transient delay of escape might highlight a rather indirect impact of NrampB, which is located at the contractile vacuole, in contrast to a direct impact of Nramp1 which is present at the *F.n.n*. vacuole.

### *F.n.n*. iron transporters are upregulated in the *Dictyostelium nramp* mutants

In principle, intracellular bacteria respond to limiting iron concentrations in their environment by upregulating the transcription of genes encoding for iron transporters (Rodriguez et al., 2002; Deng et al., 2006; Ledala et al., 2010). *F.n.n*. possesses two iron accumulation systems: the Feo system for uptake of soluble Fe^2+^, represented by FeoA and FeoB, and the siderophor synthetase IucA/C for transport of insoluble Fe^3+^. Members of both uptake systems (*feoA, iucA/C*) showed highly increased mRNA levels after depletion of iron during *in vitro* growth of *F.n.n*. (Figure S3).

**Figure 4:**
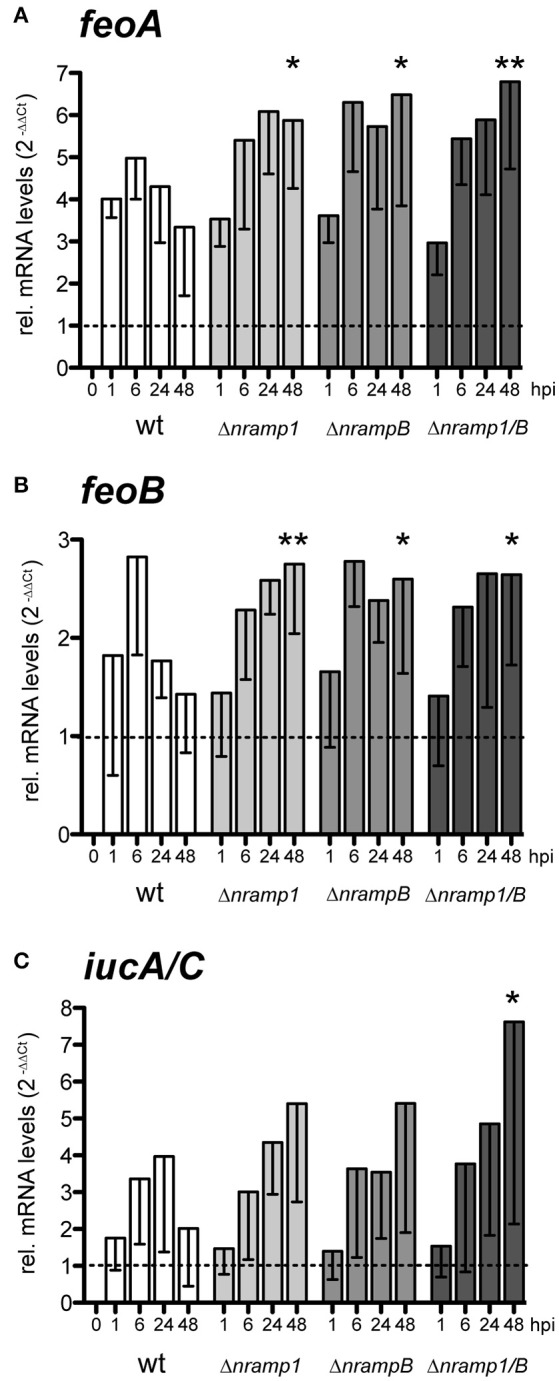
Relative mRNA levels of *F.n.n*. iron associated genes during infection of *Dictyostelium* wt and *nramp* mutant cells. **(A–C)** *Dictyostelium* wt, Δ*nramp1*, Δ*nrampB*, and Δ*nramp1/B* cells were infected with *F.n.n*. wt GFP and mRNA amounts of *F.n.n. feoA* **(A)**, *feoB* **(B)**, and *iucA/C* **(C)** were measured by qRT-PCR over 48 hpi. *In vitro* cultivated *F.n.n*. supplemented with 2 mM FeCl_3_ served as a mock control (dotted line). For each timepoint, *nramp* mutants were compared to the wt using a repeated measures one-way ANOVA with Dunnett's *post-hoc* analysis: ^*^p < 0.05, ^**^p < 0.01.

We monitored mRNA levels of the iron accumulation genes *feoA, feoB* and *iucA/C* during infection of wt cells and normalized it to cultured bacteria (dotted line; Figures 4A–C). At 6 hpi, we observed an induction of all three genes, when most of the bacteria are entering the cytosolic growth phase. At later timepoints, mRNA levels of the entire gene set were still elevated in comparison to *in vitro* cultured bacteria but gradually decreased until 48 hpi. These observations suggest that *F.n.n*. relies on the uptake of external iron during the cytosolic growth phase in *Dictyostelium* cells.

Peracino et al. (2013) observed that *nramp* mutants contain lower levels of intracellular, bioavailable iron. Accordingly, *F.n.n*. showed an increased induction of all iron related genes in the late infection phase of the *nramp* mutants compared to wt cells. Together, these results suggest that cytosolic *F.n.n*. experience an enhanced iron limitation in the absence of Nramp1 and NrampB, to which the bacteria respond by an upregulation of iron transporters.

### Phagosomal maturation profiles are altered in *nramp* mutants

Nramp1 is known to play a role in the maturation of phagosome-derived, pathogen-containing compartments during infection with bacteria such as *Salmonella* and *Mycobacteria* (Hackam et al., 1998; Govoni et al., 1999; Frehel et al., 2002). In *Dictyostelium*, phagosomal maturation is characterized by rapid lowering of the pH and delivery of proteolytic enzymes followed by the reneutralization of the compartment after 1–2 h via recycling of the V-ATPase from the phagosomal membrane (Clarke et al., 2002, 2010). To investigate an impact of Nramp1 and NrampB on phagosomal maturation in our system, we measured the phagosomal proteolysis and pH in wt and *nramp* mutant cell lines over 2 h using DQ Green BSA- (proteolysis-sensor) or FITC-labeled (pH-sensor) latex beads (Sattler et al., 2013).

**Figure 5:**
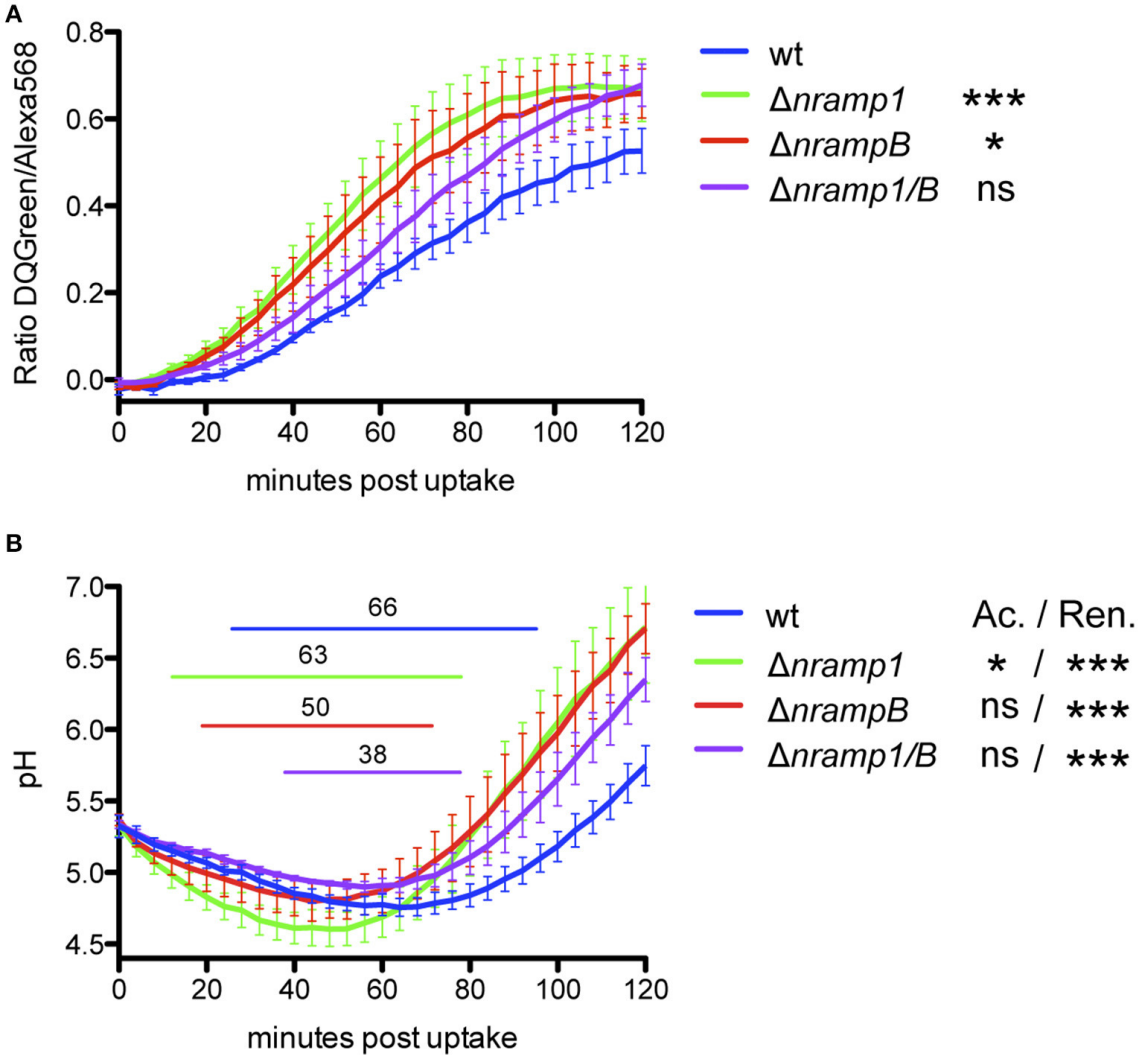
Phagosomal proteolysis and acidification profile of *Dictyostelium* wt and *nramp* knockout cell lines. To monitore proteolysis **(A)** and pH **(B)** in the phagosome, *Dictyostelium* cells took up DQGreen-BSA/AlexaFluor568- or FITC/AlexaFluor568-labeled 3 μm silica beads by phagocytosis. The fluorescence emission ratio at 520/595 nm of intracellular beads was measured every 4 min for 2 h. **(A,B)** For statistical analysis of proteolysis and pH profiles of *nramp* mutants to wt cells, the slopes of the linear graph ranges were calculated and tested for significance using a one-way ANOVA with Dunnett's *post-hoc* test: ^*^p < 0.05, ^**^p < 0.01, ^***^p ≤ 0.001. Elevated lines in **(B)** indicate the starting point and duration of pH < 5 for each cell line. Displayed is the mean ± SEM of four independent experiments. Ac: Acidification, Ren: reneutralization.

After phagocytosis of DQ Green BSA-labeled beads, we observe a significantly faster and stronger bulk proteolysis in *nramp* single mutants compared to *Dictyostelium* wt cells (Figure 5A, Figure S4). The double mutant shows an intermediate phenotype between wt and single mutant cell lines. All cell lines show an immediate decrease of the pH in the FITC-labeled bead containing phagosomes, which is significantly stronger in Δ*nramp1* compared to wt cells (Figure 5B, Figure S4). However, *nramp* mutants differ slightly in starting point and duration of acidification represented by a pH below 5 (indicated by the elevated line). An earlier (Δ*nramp1*) or shorter (Δ*nrampB*, Δ*nramp1/B*) acidification below pH 5 coincided with a significantly faster reneutralization of the latex bead-containing phagosome compared to wt cells. Together, these results indicate an impact of the Nramp transporters on phagosomal maturation of inert particles.

## Discussion

**Figure 6:**
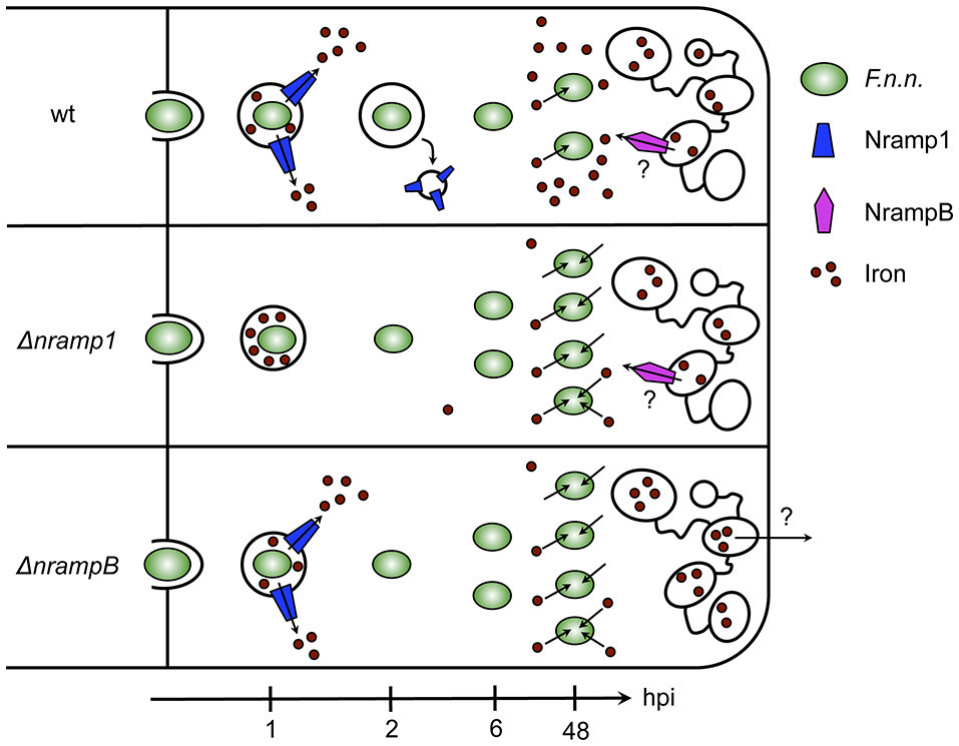
Model of *F.n.n*. infection course in *Dictyostelium* wt, Δ*nramp1*, and Δ*nrampB* cells over 48 hpi. In wt cells, Nramp1 associates with the *F.n.n*.-phagosome but is removed from the compartment after 2 hpi dependent on IglC. At 6 hpi, most bacteria escaped the phagosome into the cytosol where they upregulate bacterial iron transporters for ferrous and ferric iron and start replicating. In *nramp knockout* cells, *F.n.n*. reaches its replicative niche earlier and more efficiently than in wt cells and subsequently more bacteria contribute to the bacterial burden. The bacteria respond to the lower cellular iron levels of the *nramp* mutants by an increased upregulation of iron uptake factors. NrampB is localized exclusively at the contractile vacuole and might therefore control phagosomal maturation and *F.n.n*. escape from the phagosome through regulation of global cellular iron homeostasis.

In this study, we showed that the iron transporters Nramp1 and NrampB contribute to resistance against *Francisella* in the *Dictyostelium*/*F.n.n*. model system. In mutants lacking either one or both of the Nramp transporter *F.n.n*. escaped its phagosome more efficiently resulting in higher bacterial burdens (summarized in Figure 6).

Iron is an essential nutritional factor for both the host and its pathogen and is tightly regulated by them during infection. This need for iron can also be used as a defense strategy against infection. For example, mammals can react against a bacterial infection by lowering serum iron levels (hypoferremia), which is also observed in humans during infection with *F. tularensis* (Pekarek et al., 1969; Kim et al., 2014).

The host iron transporter Nramp1 regulates iron homeostasis on the cellular level and contributes to resistance against vacuolar pathogens, like *Mycobacteria, Legionella* and *Leishmania* (Vidal et al., 1993, 1995; Frehel et al., 2002; Fritsche et al., 2012). Accordingly, in *Dictyostelium*, Nramp1 and NrampB also contribute to resistance against *Mycobacteria* and *Legionella* infection (Peracino et al., 2006, 2010, 2013).

Nramp function has only been investigated for bacteria thriving in vacuoles and the mechanism is still unknown. In general, two mechanisms are proposed to limit bacterial growth: the depletion of nutritional iron from the bacterial vacuole (Soldati and Neyrolles, 2012; Bozzaro et al., 2013) and to antagonize bacterial virulence strategies that are meant to block bactericidal activity in the phagosome (Hackam et al., 1998; Frehel et al., 2002; Cellier et al., 2007; Fritsche et al., 2012). The role of Nramps for cytosol-dwelling bacteria has not been well characterized on the cellular level. Therefore, we describe here the role of Nramp1 and NrampB for *Francisella* infection in the established *Dictyostelium*/*F. noatunensis* subsp. *noatunensis* model (Lampe et al., 2016).

We observed the transient recruitment of Nramp1 to the *F.n.n*. phagosome with a peak in association at 1 hpi followed by a fast decline. In contrast, the phagosome of avirulent *F.n.n*. Δ*iglC* bacteria remained Nramp1-positive until 2 hpi indicating a (direct or indirect) role of the type 6 secretion system for Nramp1 association with the phagosome during its maturation. In principle, this early loss of the membranous transporter could be caused by phagosomal escape of wt bacteria. However, the association of *F.n.n*. wt with the phagosomal marker p80 remains stable between 1 and 2 hpi (Lampe et al., 2016), therefore cytosolic translocation of wt bacteria is unlikely to lead to the observed drop in Nramp1 association. It rather suggests an active retrieval of Nramp1 by *F.n.n*.

In contrast, *Legionella* pursues a different strategy in *Dictyostelium* to manipulate Nramp1 function. Nramp1 remains at the *Legionella* compartment until 24 hpi (Peracino et al., 2010). Peracino et al. suggest that *Legionella* reverses the transport direction of the iron transporter, thereby retaining essential iron in its replication niche. In contrast to Nramp1, NrampB was never observed at the *F.n.n*. phagosome suggesting that it has no direct effect on the ion composition of the bacterial compartment.

Using microscopy and flow cytometry, we observed increased bacterial loads in the late infection phase of both *Dictyostelium nramp* mutants. This shows that the Nramp transporters contribute to resistance against *Francisella* growth in *Dictyostelium*. Similarly, *L. pneumophila* and *M. avium* show increased bacterial growth in the absence of Nramp1 and, in case of *L. pneumophila*, NrampB.

As described, the infection with *Francisella* follows two phases, a phagosomal followed by a replicative, cytosolic stage. To determine if Nramp activity has an impact on the phagosomal stage we compared *F.n.n*. virulence in the phagosome between wt and *nramp* mutant cells by monitoring their phagosomal escape using the marker p80. We observed significantly higher escape rates in the absence of Nramp1 and NrampB suggesting that Nramp activity suppresses or delays phagosomal escape. As a result, more *F.n.n*. bacteria gain access to the cytosolic replication phase in the *nramp* mutants and contribute to *F.n.n*. growth. In accordance with our observation, *M. tuberculosis* ruptures the phagosome and gains access to the cytosol more efficiently in Nramp1-deficient macrophages (Simeone et al., 2015).

Mechanistically, the intraphagosomal environment, which is actively controlled by the pathogen has been suggested to influence the translocation rate of bacteria (Beauregard et al., 1997; Chong et al., 2008; Santic et al., 2008; Napier et al., 2012; Simeone et al., 2015). In accordance, during the infection of macrophages with *Mycobacteria* and *Salmonella*, Nramp1 promotes acidification and fusion with endosomal and lysosomal vesicles thereby generating a bactericidal environment for the pathogenic bacteria (Hackam et al., 1998; Govoni et al., 1999; Frehel et al., 2002; Jabado et al., 2003). In *Salmonella* infection, this effect could be replicated in Nramp1-/- murine macrophages by using membrane-permeant iron chelators (Jabado et al., 2003). This led the authors to hypothesize that iron deprivation of the phagosome by Nramp1 counteracts the ability of the pathogen to manipulate phagosomal maturation and execute its virulence program. This is supported by studies in macrophages which showed no impact of Nramp1-deletion on phagosomal maturation of inert particles such as latex beads, non-pathogenic *Bacillus subtilis*, or dead *Mycobacteria*, but only for living pathogenic bacteria (Hackam et al., 1998; Frehel et al., 2002).

To investigate an impact of the Nramps on phagosomal maturation in our model system, we monitored the pH and proteolysis in the phagosomes of *nramp* mutants via fluorescent bead analysis and compared it to wt cells. Our results showed only minor effects of the Nramps activity on the acidification profiles but a faster and stronger proteolysis inside the phagosome. The accelerated phagosomal maturation in the *nramp* mutants might trigger virulence strategies of the bacteria leading to their escape from the phagosome. Accordingly, several studies showed that phagosomal acidification of the *F. tularensis* phagosome is important for phagosomal escape (Chong et al., 2008; Santic et al., 2008; Clemens et al., 2009), whereas Clemens *et al*. observed no impact (Clemens et al., 2009). However, while our assay was performed with inert particles, living bacteria might encounter a different phagosomal maturation profile in the *nramp* mutants. Additionally, altered phagosomal maturation had no effect on cellular growth of *nramp* mutants in axenic medium and on non-pathogenic *Klebsiella* bacteria (Lelong et al., 2011), hence, the impact of these results on living *F.n.n*. should be interpreted with caution.

Besides a specific role of Nramp activity at the phagosome in our model system, a disturbed cellular iron homeostasis in the *nramp* mutants might be responsible for the early escape of *F.n.n*. from the phagosome. *Nramp* mutants demonstrate lower levels of bioavailable iron (Peracino et al., 2013), hence the cytosolic iron content of the host cell might be responsible for an early escape of the pathogen. This is supported by the increased *F.n.n*. translocation and similar phagosomal maturation of inert particles in both *nramp* knockout cell lines although NrampB is not localized at the phagosome. Additionally, the absence of an additive effect in the *nramp* double deletion mutant implies a common mechanism.

Upon access to the cytosol of *Dictyostelium* wt cells, *F.n.n*. upregulated gene transcription of its iron accumulation systems for Fe^2+^ (*feoA, feoB*) and Fe^3+^ (*iucA/C*). This indicates that iron, which *F.n.n*. needs for intracellular growth, is limited in the cytosol of *Dictyostelium. F.n.n*. is able to grow efficiently in the *nramp* mutants despite their decreased levels of bioavailable iron. This is accompanied by an increased upregulation of the iron accumulation genes in the late phase of infection. It would be interesting to monitor bacterial growth in *Dictyostelium* cells without external iron from the growth medium, however, dramatically decreased iron concentrations disturb basic cellular processes of the amoeba (Peracino et al., 2013).

Our results are consistent with a recent study (Powell and Frelinger, 2017) showing a critical role for Nramp1 during *F. tularensis* LVS infection *in vitro* in BMDMs and *in vivo* during pulmonary infection. In the early phase of infection (1 and 4 hpi), less bacteria were observed in BMDMs with a functional Nramp1 compared to BMDMs which express a non-functional Nramp1 protein. This correlated with an increased production of ROS in the presence of functional Nramp1. Additionally, less bacterial growth occurred in Nramp1^+^ BMDMs. *In vivo* studies revealed Nramp1^+^ B6 mice resistant to intranasal infection, however not for intradermal infection, and confirmed Nramp1 as a resistance factor for *Francisella* infection. Together, this indicates a protective role for Nramp1 during the early, phagosomal stage of *Francisella* similar to our results.

Our results stand in contrast to studies by Kovarova et al., who observed less *Francisella* growth in Bcg(s) mice lacking a functional Nramp1 protein (Kovarova et al., 2000). However, Fritsche et al. showed in RAW264.7 macrophages that Nramp1 modulates the expression of other iron transporters, which results in an increased iron content of cells without a functional Nramp1 (Fritsche et al., 2007). This additional iron source could boost *Francisella* growth in the cytosol and account for the contrasting phenotype in Bcg(s) mice.

Taken together with this study we highlight the ease with which the *Dictyostelium* system allows to dissect the role of host factors in *Francisella* infection. We show that Nramp-transporters protect the host cell from increased *Francisella* growth. Most importantly, Nramp1 or NrampB contribute to host resistance against *Francisella* infection rather by reducing the bacteria's translocation efficiency to their replicative niche than by restriction of nutritional iron in the cytosol.

## Author contribution

YB, HW, and MH designed experiments and wrote the manuscript. YB and DO performed experiments.

### Conflict of interest statement

The authors declare that the research was conducted in the absence of any commercial or financial relationships that could be construed as a potential conflict of interest.

## Supplementary material

Figure S1 Survival test of exocytosed *F.n.n*. wt and Δ*iglC*. Supernatant of *Dictyostelium* cell cultures infected with *F.n.n*. wt (left) and Δ*iglC* (right) at 6 hpi was tested for living *F.n.n*. on chocolate agar plates. Undiluted and 1:50 diluted bacterial suspensions were used (*n* = 2).

Figure S2 Cell growth of *Dictyostelium* wt and *nramp* knockout cell lines during *F.n.n*. infection over 48 hpi (*n* = 7 ± SEM).

Figure S3 Relative mRNA levels of *F.n.n*. iron uptake factors FeoA and IucA/C under iron limitation *in vitro*. Iron was sequestered in the *F.n.n*. growth medium via the iron chelator 2,2′-dipyridyl. *F.n.n*. grown with 2 mM FeCl_3_ were used as a mock control. (*feoA*: *n* = 4, *iucA/C*: *n* = 2).

Figure S4 Linear regression of the range of interest for the phagosomal proteolysis and pH profiles. **(A)** To compare bead-phagosome proteolysis of *nramp* mutant cell lines to wt cells, a linear regression function was calculated for the linear range of each cell line's proteolysis profile and displayed in the corresponding color. **(B,C)** Acidification **(B)** and Reneutralization **(C)** of bead-containing phagosomes were compared for each cell line using the slope of the linear regression of *t* = 0 min to the minimal turning point (mtp) **(B)** and of the mtp to *t* = 120 min **(C)**. Ac: acidification, Ren: reneutralization. Slopes were tested for significance differences using a one-way ANOVA with Dunnett's *post-hoc* analysis. ^*^*p* < 0.05, ^***^*p* < 0.001.

Table S1 Primer sequences of *F.n.n*. reference and iron accumulation genes.
