# Supplementary figures and images for: Nramp1 and NrampB Contribute to Resistance against Francisella in Dictyostelium
Source: Front Cell Infect Microbiol. 2017 Jun 21;7:282. doi: 10.3389/fcimb.2017.00282 (PMC5478718; doi:10.3389/fcimb.2017.00282)

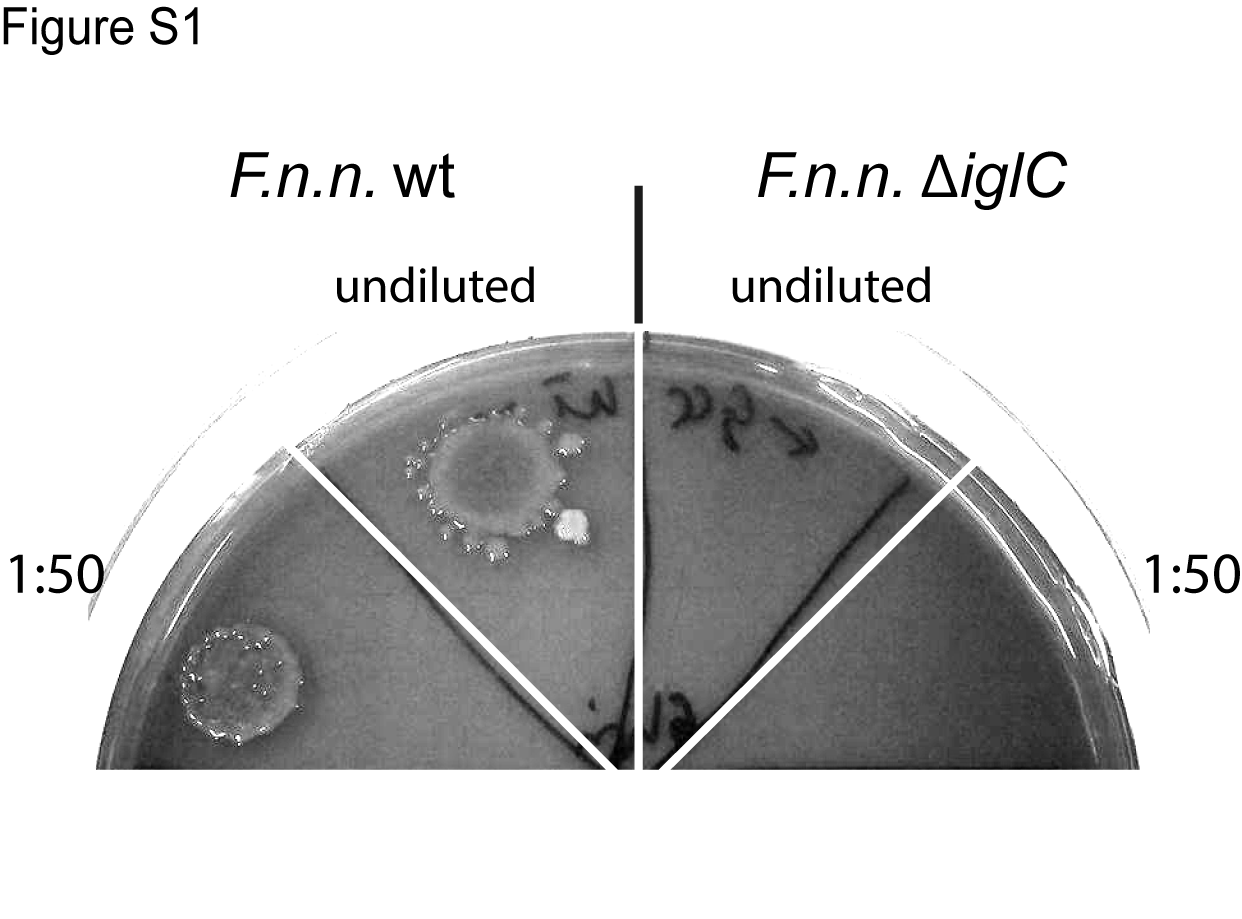

Supplement: Figure S1 — Survival test of exocytosed F.n.n. wt and ΔiglC. Supernatant of Dictyostelium cell cultures infected with F.n.n. wt (left) and ΔiglC (right) at 6 hpi was tested for living F.n.n. on chocolate agar plates. Undiluted and 1:50 diluted bacterial suspensions were used (n = 2). [file Image1.TIF]

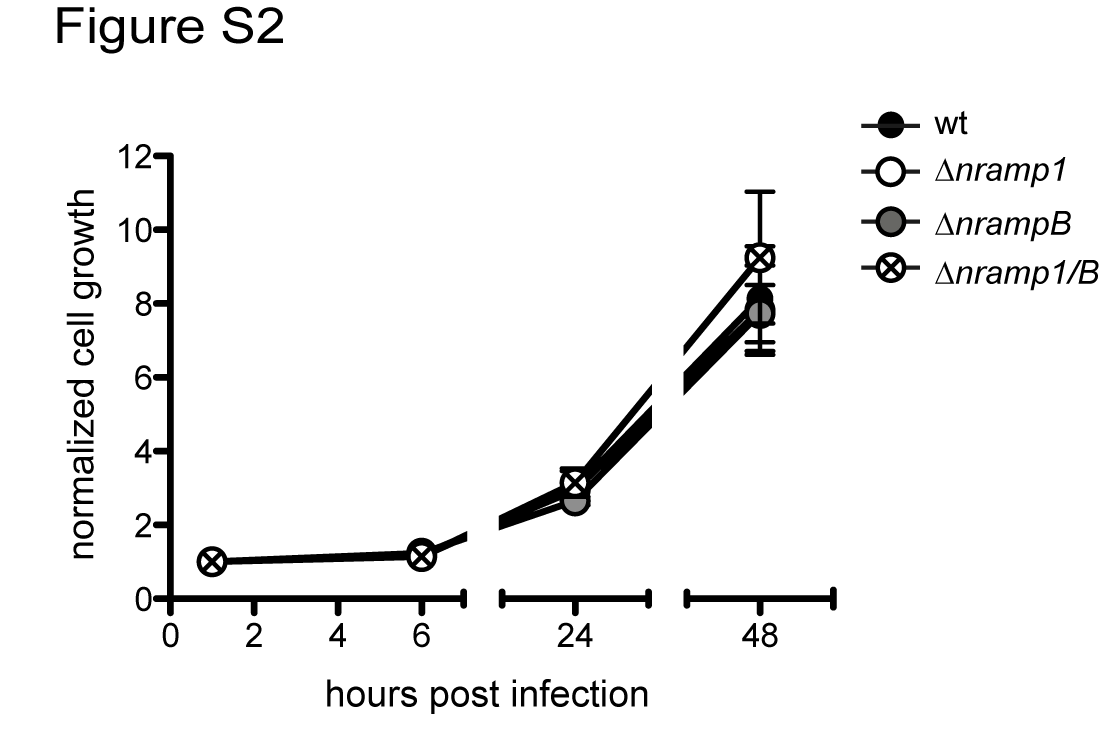

Supplement: Figure S2 — Cell growth of Dictyostelium wt and nramp knockout cell lines during F.n.n. infection over 48 hpi (n = 7 ± SEM). [file Image2.TIF]

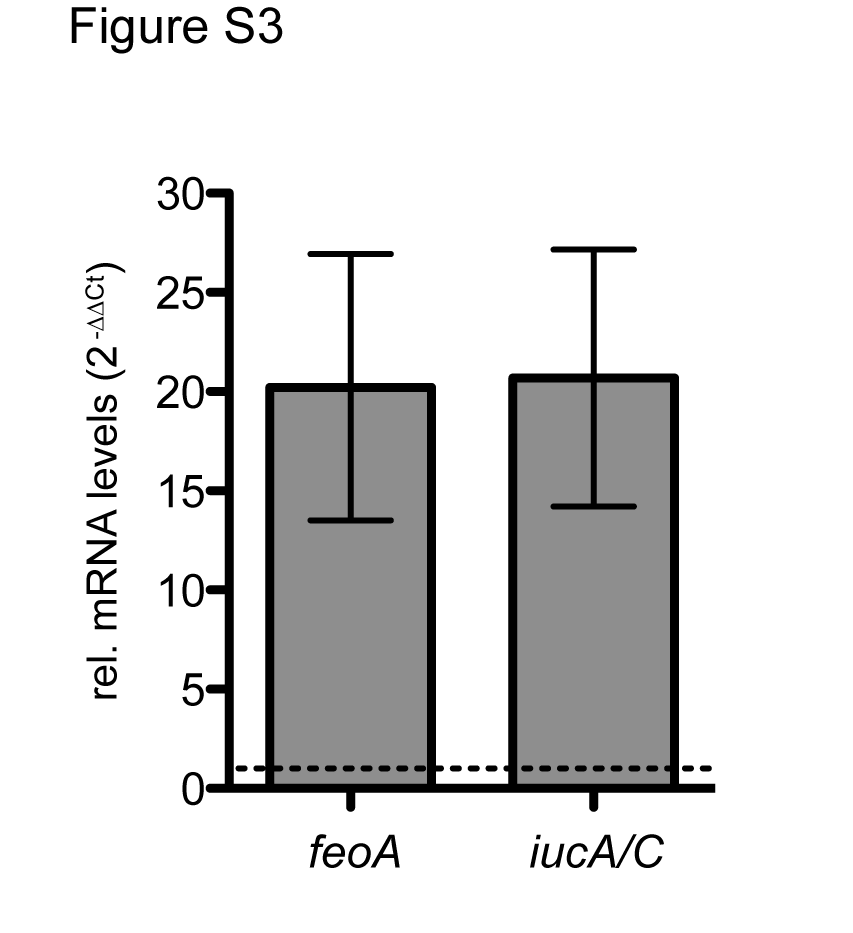

Supplement: Figure S3 — Relative mRNA levels of F.n.n. iron uptake factors FeoA and IucA/C under iron limitation in vitro. Iron was sequestered in the F.n.n. growth medium via the iron chelator 2,2′-dipyridyl. F.n.n. grown with 2 mM FeCl3 were used as a mock control. (feoA: n = 4, iucA/C: n = 2). [file Image3.TIF]

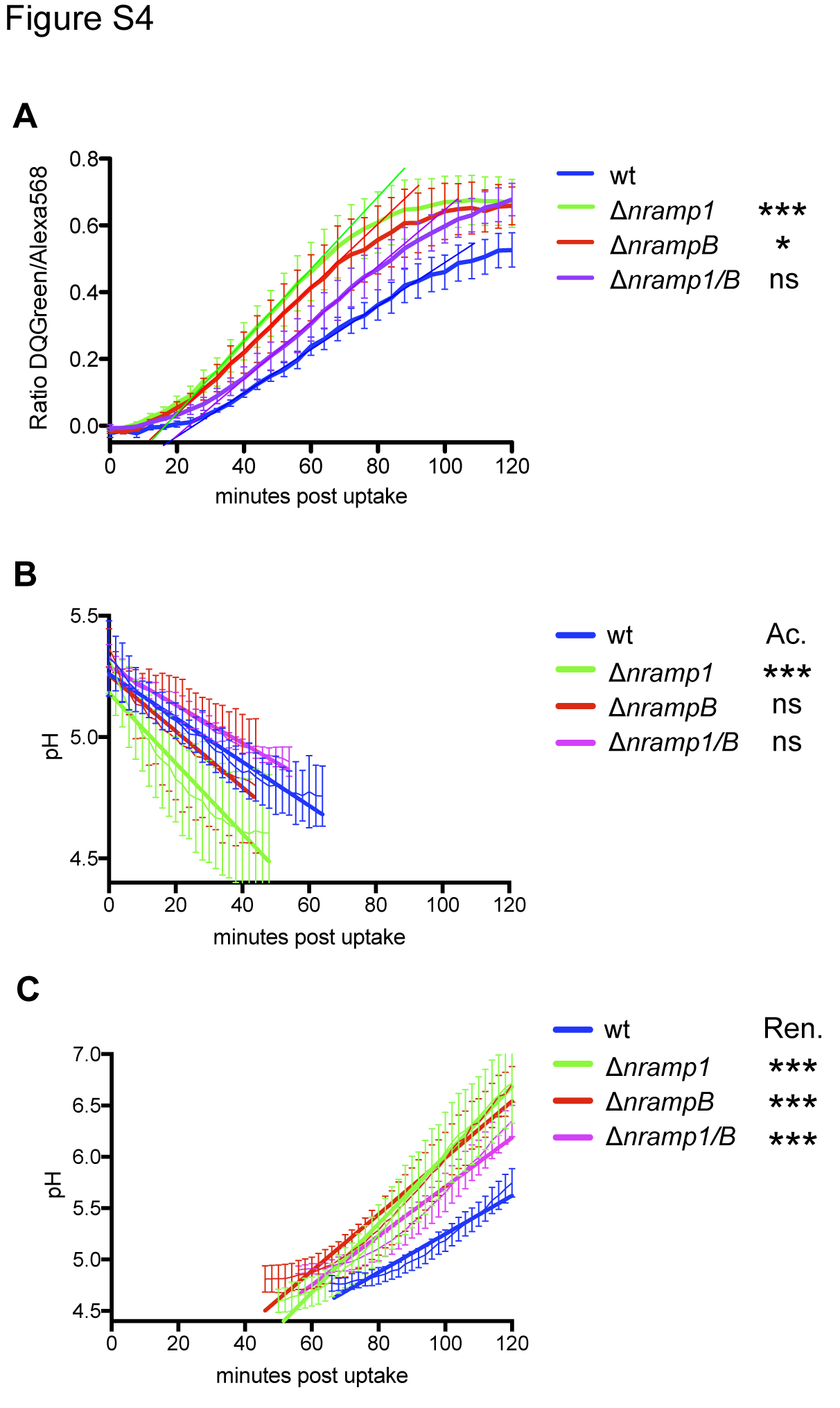

Supplement: Figure S4 — Linear regression of the range of interest for the phagosomal proteolysis and pH profiles. (A) To compare bead-phagosome proteolysis of nramp mutant cell lines to wt cells, a linear regression function was calculated for the linear range of each cell line's proteolysis profile and displayed in the corresponding color. (B,C) Acidification (B) and Reneutralization (C) of bead-containing phagosomes were compared for each cell line using the slope of the linear regression of t = 0 min to the minimal turning point (mtp) (B) and of the mtp to t = 120 min (C). Ac: acidification, Ren: reneutralization. Slopes were tested for significance differences using a one-way ANOVA with Dunnett's post-hoc analysis. *p < 0.05, ***p < 0.001. [file Image4.TIF]
