# Supplementary material for: Nramp1 and NrampB Contribute to Resistance against Francisella in Dictyostelium
Source: Front Cell Infect Microbiol. 2017 Jun 21;7:282. doi: 10.3389/fcimb.2017.00282 (PMC5478718; doi:10.3389/fcimb.2017.00282)
Supplement: Table S1 — Primer sequences of F.n.n. reference and iron accumulation genes. [file Table1.DOCX]

Table 1: Primer sequences of *F.n.n.* reference and iron accumulation genes

| Gene | Primer | Sequence (5’-3’) |
| --- | --- | --- |
| *ftsZ* | ftsZ_forward | TACCATACTCAGCGGCTTTC |
|  | ftsZ_reverse | GCGCCTGTAGTTGCTGAAGT |
| *polA* | polA_forward | AGCTGGAACTGGTCGTAATCA |
|  | polA_reverse | ATCAGCATCTTCAGCAGCATA |
| *fopA* | fopA_forward | AGAGATGTTCAGGTGAGTGC |
|  | fopA_reverse | ATGTTAGTACCTGCTCTACCC |
| *feoA* | feoA_forward | TTCATAATTGTCCTGTCGCATAC |
|  | feoA_reverse | CATTTCTCACACTGACTTGGC |
| *feoB* | feoB_forward | CTTTTCAACATGGACCTAAACCC |
|  | feoB_reverse | TGTCATATTACCCATAGCAGAGC |
| *iucA/C* | iucA/C_forward | ACCCCTATACTTTGGCTGTACT |
|  | iucA/C_reverse | TCCTCAGATTCTCCACATAGACT |
